# Supplementary material for: Gold and Iron Oxide Nanoparticle Assemblies on Turnip Yellow Mosaic Virus for In-Solution Photothermal Experiments
Source: Nanomaterials (Basel). 2023 Sep 7;13(18):2509. doi: 10.3390/nano13182509 (PMC10535558; doi:10.3390/nano13182509)
Supplement: Supplementary file 1 [file nanomaterials-13-02509-s001.zip › nanomaterials-2571295-supplementary.pdf]

Supporting information

Gold and iron oxide nanoparticles assemblies on Turnip yellow mosaic virus for in solution photothermal experiments

by

Ha Anh Nguyen [1,2], Sendos Darwish [2], Hong Nam Pham [3], Souad Ammar [2] and Nguyet-Thanh Ha-Duong [2,*]

The X-ray diffraction (XRD) patterns of the synthesized IONP before and after citrate functionalization (Figure S1) were recorded using ~~with~~ a Panalytical X'Pert Pro diffractometer equipped with Co Kα (λ = 1.789 Å, 40 kV, 40 mA) radiation. The cell parameter and the average crystallite size of the coherent diffraction domain were determined with MAUD software. MAUD [1] is based on the Rietveld method combined with Fourier analysis which is well-adapted for broad diffraction peaks. In both cases, they matched very well with the spinel structure, with a cell parameter of 8.36 ± 0.02 Å and an average crystallite size of 9.4 ± 1.5 nm.

Attenuated Total Reflection Infrared (ATR-IR) Spectroscopy was carried on a Magna 860 device composite on a Fourier transform infrared (FT-IR) spectrometer. Generally, a drop of sample was dried on the diamond prism using a hair blower, which is capable of analyzing samples in the mid-infrared range (4000-400 cm^{-1}). The collected spectra confirm the functionalization of IONP by citrate (Figure S1B, black). The broad band observed between 3000 cm^{-1} and 3400 cm^{-1} indicates the presence of water and/or polyol adsorbed on the as-produced iron oxide particles as well as the OH groups originated from citrate in the coated ones. The Fe-O vibration band at 586 cm^{-1} was also observed for the two types of particles. The comparison of the spectra of these particles also evidences differences. After functionalization the bands corresponding to the symmetric and asymmetric C=O stretching vibration around 1300 and 1500 cm^{-1}, appear as more intense, with a shift for the former band from 1379 cm^{-1} for pristine particles to 1394 cm^{-1} for functionalized ones, confirming the attachment of citrate on IONPs [2].

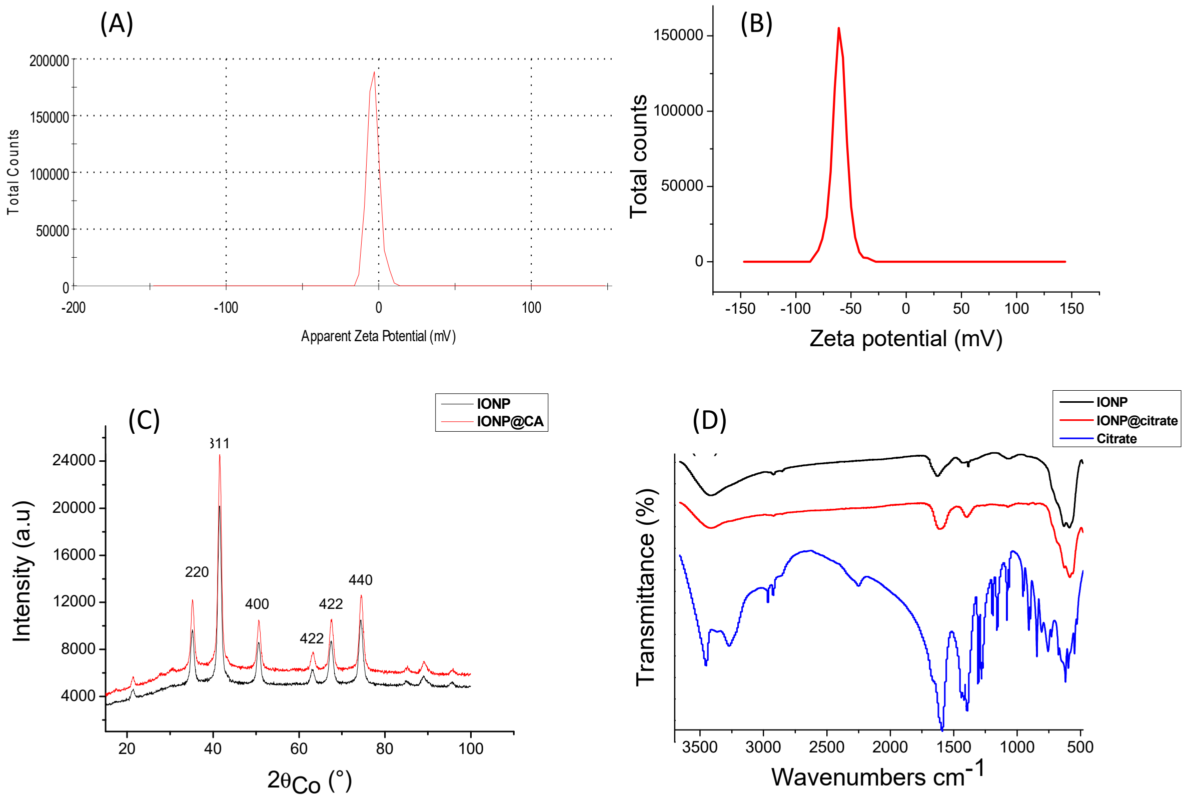

Figure S1. Characterizations of IONP and IONP@citrate. Zeta potential of IONP (A) and IONP@CA (B). (C) XRD diffractograms (D) IR spectra

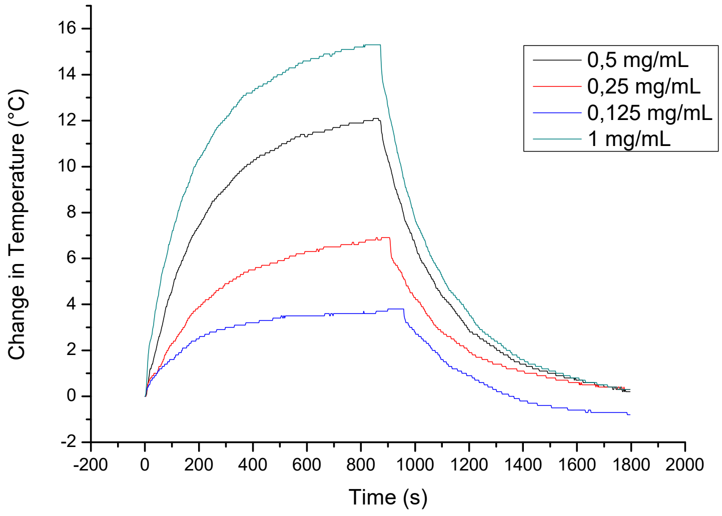

Figure S2. Temperature curve of IONP aqueous colloids at different iron concentrations under laser radiation at 808 nm (0.41 W/cm^2)

[1] Lutterotti, L.; Matthies, S.; Wenk, H. A friendly Java program for material analysis using diffraction, *CPD Newsl.*, **1999**, *21*, 14-15.

[2] ur Rahman, Z.; Dong, Y.L.; Ren, C.; Zhang, Z.Y.; Chen, X. Protein adsorption on citrate modified magnetic nanoparticles. *J. Nanosci. Nanotechnol.,* **2012**, *12*, 2598-2606.
